# HOSPITAL VOLUME, POSTOPERATIVE MORTALITY, AND COSTS AFTER GASTRECTOMY FOR GASTRIC CANCER IN COLOMBIA: IS THERE ANY ASSOCIATION?

**DOI:** 10.1590/0102-672020230027e1745

**Published:** 2023-07-10

**Authors:** Antonio Jose Cuesta, Oscar Guevara, Giancarlo Buitrago

**Affiliations:** 1 Universidad Nacional de Colombia, Faculty of Medicine, Department of Surgery – Bogotá, Colombia; 2 Hospital Universitario Nacional de Colombia – Bogotá, Colombia.

**Keywords:** Stomach neoplasms, Survival, Health care costs, Neoplasias gástricas, Sobrevida, Custos de cuidados de saúde

## Abstract

**BACKGROUND:**

There are no information in the literature associating the volume of gastrectomies with survival and costs for the health system in the treatment of patients with gastric cancer in Colombia.

**AIMS:**

The aim of this study was to analyze how gastrectomy for gastric cancer is associated with hospital volume, 30-day and 180-day postoperative mortality, and healthcare costs in Bogotá, Colombia.

**METHODS:**

A retrospective cohort study based on hospital data of all adult patients with gastric cancer who underwent gastrectomy between 2014 and 2016 using a paired propensity score. The surgical volume was identified as the average annual number of gastrectomies performed by the hospital.

**RESULTS:**

A total of 743 patients were included in the study. Hospital mortality at 30 and 180 days postoperatively was 36 (4.85%) and 127 (17.09%) patients, respectively. The average health care cost was USD 3,200. A total of 26 or more surgeries were determined to be the high surgical volume cutoff. Patients operated on in hospitals with a high surgical volume had lower 6-month mortality (HR 0.44; 95%CI 0.27–0.71; p=0.001), and no differences were found in health costs (mean difference 398.38; 95%CI–418.93–1,215.69; p=0.339).

**CONCLUSIONS:**

This study concluded that in Bogotá (Colombia), surgery in a high-volume hospital is associated with better 6-month survival and no additional costs to the health system.

## RESUMO

**RACIONAL:**

Não há informações na literatura relacionando o volume de gastrectomias bem como a sobrevida e os custos para o sistema de saúde, no tratamento de pacientes com câncer gástrico na Colômbia.

**OBJETIVOS:**

analisar como a gastrectomia para câncer gástrico está associada ao volume hospitalar, mortalidade pós-operatória de 30 e 180 dias e custos de saúde em Bogotá, Colômbia.

**MÉTODOS:**

Estudo de coorte retrospectivo baseado em dados hospitalares de todos os pacientes adultos com câncer gástrico submetidos à gastrectomia entre 2014 e 2016, utilizando um escore de propensão pareado. O volume cirúrgico foi identificado como o número médio anual de gastrectomias realizadas pelo hospital.

**RESULTADOS:**

Foram incluídos no estudo 743 pacientes. A mortalidade hospitalar aos 30 e 180 dias de pós-operatório, foram respectivamente, 36 (4,85%) e 127 (17,09%) pacientes. O custo médio de saúde foi de US$ 3.200. Vinte e seis ou mais cirurgias foram determinadas como ponto de corte de alto volume cirúrgico. Pacientes operados em hospitais de alto volume cirúrgico tiveram menor mortalidade em seis meses (HR 0,44; IC95% 0,27–0,71; p=0,001) e não foram encontradas diferenças nos custos com saúde (diferença média 398,38; IC95% −418,93–1215,69; p=0,339).

**CONCLUSÕES:**

Este estudo concluiu que em Bogotá (Colômbia), a cirurgia em um hospital com alto volume cirúrgico está associada a uma melhor sobrevida de seis meses e não há custos adicionais para o sistema de saúde.

## INTRODUCTION

The surgical treatment of patients with gastric cancer is a key element in their prognosis, particularly for early or locally advanced stages^1,16^. However, many patients are not detected by the health system until after the disease has reached advanced stages, especially in low-income countries. Consequently, gastrectomies for gastric cancer patients are highly complex procedures that require the development of skills and abilities not only by healthcare professionals but also by the institutions that perform the surgeries^21^.

Discussions have occurred in various health systems about centralizing specialized procedures in hospitals that have a high surgical volume for cancer treatment. The staff in hospitals with a high surgical volume has more training in these surgeries and in managing complications, using technology based on patient needs, and applying standardized processes that can improve outcomes. However, these specialized centers could generate more health system costs due to the high cost of their skilled staff, the technology they use, and the need to transport patients to these centers^6,7,20,24^.

Colombia, a middle-income country, has a mandatory health system with managed-care competition, where public and private insurers provide a standard benefit package for all enrollees. The benefit package includes treatment for gastric cancer. The healthcare system is divided into a contributory system for formal workers and their families and a subsidized system for low-income families. These two systems cover 96% of Colombians. Bogota, the capital of Colombia, is home to 18% of the Colombian population (approximately 9 million residents), and its service delivery network is composed of hospitals that are very similar in size, complexity, and experience. The different hospitals treat gastric cancer patients in accordance with the contracts between the insurers and providers. Therefore, Bogota is an ideal location to evaluate the relationship between hospital volume and clinical and financial outcomes related to gastric cancer patients who undergo gastrectomies in resource-limited settings.

There is no information from low-income countries about how surgical volume in hospitals is related to survival and the costs that health systems incur when treating gastric cancer patients.

This study was aimed at analyzing how a gastrectomy for gastric cancer is associated with hospital volume, 30-day and 180-day postoperative mortality, and healthcare costs in Bogota, Colombia.

## METHODS

### Study design and data sources

This is a retrospective cohort study based on hospital data, using the UPC (capitation payment unit) sufficiency database from the Colombian Ministry of Health and Social Protection and death certificates from the Unique Enrollment Registry (RUAF in Spanish) in Bogota. All patients in the contributory and subsidized systems who were diagnosed with gastric cancer and who underwent a gastrectomy from January 2014 to December 2016 were included. All patients were followed up to 6 months after surgery.

The UPC database contains information that Colombia's health system insurers send to the Ministry of Health, which is used to estimate the premiums that the system recognizes for each person enrolled in the health system. This database is highly standardized and contains detailed information about all services used by the enrollees, including the type of service provided, the International Code of Diseases-10 (ICD-10), date of service, municipality, sex, age, insurer, service provider, and the cost paid by each insurer (the cost incurred by the health system for each enrollee). The RUAF death certificate database contains information on all deaths in the country and includes the date and cause of death. Death certificates in Colombia have a coverage rate of over 90%^22^. The databases were anonymized, and the study complied with the standards of the Declaration of Helsinki and the current ethical guidelines and was approved by the Institutional Review Board of the School of Medicine at the National University of Colombia (number 023-264, on 11/23/2019).

### Variables

The primary exposure variable was the average annual volume of gastrectomies performed at the hospital at which the patient underwent a gastrectomy. Two groups were identified:

1. patients who were treated at hospitals with a high surgical volume (the exposed cohort) and
2. patients who were treated at hospitals with a low surgical volume (the unexposed cohort).

To divide the population into exposures to high and low volumes, different cutoff points were tested based on the observed distribution of surgical volume. The relative risk of 30-day mortality was estimated for each possible cutoff point evaluated, and the lowest point that was statistically <1 was selected as the cutoff between high and low volumes. This relative risk was estimated with multivariate Poisson models of 30-day mortality as a function of exposure status (for each surgical volume value between 10 and 31 surgeries per year) and was adjusted for type of regime (contributory and subsidized), age, Charlson index (which includes metastatic cancer), sex, and year of surgery. Confidence intervals of 95% were used.

### Outcomes

The primary outcome was survival at 180 days after surgery. In addition, the total costs of care incurred by the health system were estimated for all health services used during the first 30 days after surgery. These costs included surgery, related hospitalization, medications, and diagnostic tests and were adjusted to 2016 USD.

### Analysis

All baseline characteristics and outcomes were described using absolute and relative frequencies for categorical variables and means and standard deviations for continuous variables. Due to the lack of randomization, propensity score matching (PSM) was used to decrease selection bias and confounding variables^4^. A logistic regression model was used to obtain the propensity score for each subject, which included clinical and demographic variables that were predictive of exposure status.

This model took into account all possible observable confounders and variables that were imbalanced in the descriptive analyses. A 1:1 nearest neighbor match without replacement was used to match subjects in high surgical volume hospitals (the exposed cohort) with subjects in low surgical volume hospitals (the unexposed cohort). Matching algorithms with different calipers were tested, and the one that produced the best balance in the baseline characteristics of the matched sample was selected. Balance was evaluated based on the absolute value of the standardized differences in the matched sample, with a target value less than 0.1^5^.

The association between surgeries in a high-volume surgical hospital and 180-day survival was estimated for the matched sample with hazard ratios (HR) using Cox models^3^. The assumption of proportionality was evaluated.

A linear regression of the matched sample was also used to determine the association between high *versus* low surgical volume (exposure status) and healthcare cost, and 95% confidence intervals (CI) with robust standard errors in matching were estimated. All analyses were performed with Stata 17 (StataCorp LP, College Station, TX).

## RESULTS

### Descriptive

A total of 743 patients with a diagnosis of gastric cancer underwent gastrectomy in Bogota from January 2014 to December 2016. Of those, 62% were women, and 30.42% belonged to the subsidized health system. The mean age was 61.70 years (SD 13.71). A total of 36 (4.85%) patients died within 30 days after surgery and 127 (17.09%) within 180 days. The health system paid an average of USD 3,200 for healthcare costs (Table 1).

**Table 1:** Baseline characteristics of the full sample.

Characteristics		Full Sample n=743
Women; n (%)		457 (61.51)
Age, years; mean (SD)		61.70 (13.71)
Age category, years; n (%)		
	49 or less	145 (19.52)
	50–64	265 (35.67)
	65–79	276 (37.15)
	80 or more	57 (7.67)
Subsidized system; n(%)		226 (30.42)
Charlson Comorbidity Index; n(%)		
	2	422 (56.8)
	3	165 (22.21)
	4	53 (7.13)
	5	59 (7.94)
	6	20 (2.69)
	7	16 (2.15)
	8 or more	8 (1.08)
Year of surgery; n(%)		
	2014	298 (40.11)
	2015	211 (28.40)
	2016	234 (31.49)
30-day mortality; n(%)		36 (4.85)
180-day mortality; n(%)		127 (17.09)
Healthcare costs, USD 2016;mean (SD)		3,200.12 (4,790.28)

SD: standard deviation; USD: United States dollars.

### Hospital surgical volume

A total of 743 patients underwent surgery in 52 high-complexity hospitals. Three of the hospitals had annual gastrectomy volumes over 20 surgeries (one with 26, one with 30, and one with 54 gastrectomies per year). Table 2 shows the proportion of 30-day and 180-day mortality according to hospital surgical volume. The hospitals with more than 20 surgeries per year had lower 30- and 180-day mortality rates than those with lower volumes.

**Table 2:** Description of hospital surgical volume and mortality outcomes.

Hospital surgical volume categories	Patients (n)	Hospitals (n)	30-day mortality (n) (%)	180-day mortality (n) (%)
1–9 gastrectomies per year	191	37	9 (4.71)	36 (18.85)
10–19 gastrectomies per year	330	12	22 (6.67)	70 (21.21)
20–29 gastrectomies per year	87	2	1 (1.15)	9 (10.34)
30 or more gastrectomies per year	135	1	4 (2.96)	12 (8.89)
Total	743	52	36 (4.85)	127 (17.09)

CI: confidence interval; RR: relative risk.

**Figure 1:**
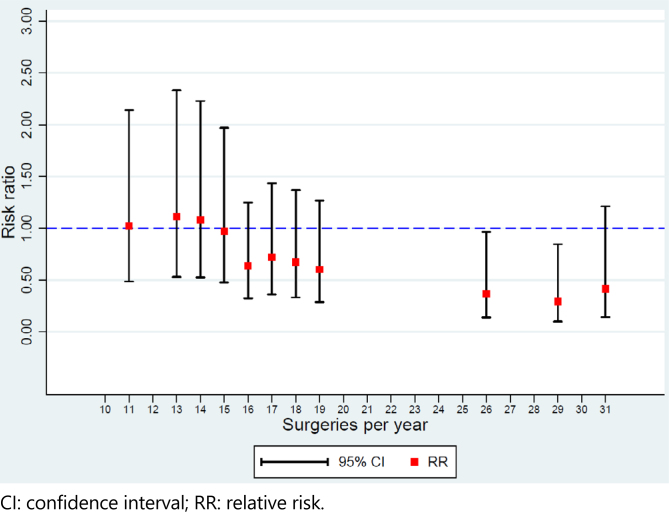
Surgical volume and 30-day mortality risk.

Different surgical volume values were tested to identify the cutoff point for dividing the sample into high volume (i.e., exposed) and low volume (i.e., non-exposed) cohorts. The adjusted relative risk of 30-day mortality was estimated for each surgical volume value tested, and the lowest value that was statistically <1 was defined as the cutoff point. Figure 1 shows the results of the analyses that were performed.

The risk of dying within 30 days was lower after 26 surgeries per year, with a relative risk of 0.37 (95%CI 0.14–0.96; p=0.04). Therefore, this was used as the cutoff point. It is important to mention that there were no hospitals with surgical volumes between 20 and 25 surgeries per year, so these values could not be tested as cutoff points for defining a high surgical volume.

### Mortality

After identifying the cutoff between high- and low-volume hospitals, the baseline characteristics of the patients were compared, and differences were observed between cohorts. Specifically, the proportion of women, age, Charlson index, and year of surgery were found to be imbalanced (absolute standardized differences greater than 0.1) (Table 3). A 1:1 propensity score matching with a caliper of 0.05 produced a good balance of baseline characteristics (absolute standardized differences less than 0.1), and the sample did not result in any significant loss in size compared to the original sample (the final size of each cohort was 221 patients).

**Table 3:** Baseline characteristics by cohort for the full and matched samples.

Characteristics		Full sample			Matched sample		
		Exposed (n=222)	Non-exposed (n=521)	Standardized differences	Exposed (n=221)	Non-exposed (n=221)	Standardized differences
Women; proportion		0.45	0.36	0.19	0.45	0.45	0.01
Age, years; mean		64.50	60.50	0.30	64.44	64.66	0.02
Subsidized system; proportion		0.27	0.32	0.11	1.27	1.30	0.06
Charlson Comorbidity Index; proportion							
	2			Ref			Ref
	3	0.20	0.23	0.07	0.20	0.21	0.02
	4	0.08	0.07	0.05	0.08	0.08	0.01
	5	0.17	0.04	0.42	0.17	0.17	0.01
	6	0.05	0.02	0.21	0.05	0.05	0.02
	7	0.05	0.01	0.24	0.05	0.04	0.03
	8 or more	0.03	0.00	0.19	0.02	0.01	0.06
Year of surgery; proportion							
	2014			Ref			Ref
	2015	0.34	0.26	0.18	0.34	0.36	0.06
	2016	0.27	0.33	0.14	0.27	0.30	0.07

Ref: Reference.

**Figure 2:**
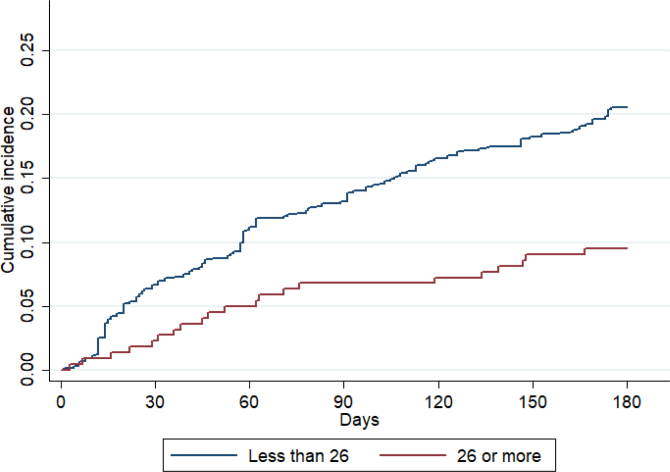
Cumulative incidence of mortality. A 6-month follow-up.

The Cox model shows that undergoing surgery in a hospital with a high surgical volume (greater than or equal to 26 surgeries per year) was associated with lower 6-month mortality (HR=0.44; 95%CI 0.27–0.71; p=0.001) for the matched sample (Table 4). Figure 2 presents the cumulative mortality curves for the two cohorts, which show a large difference between them.

**Table 4:** Effects of high volume on 6-month survival and healthcare costs.

		Estimate	95%CI	p-value
6-month survival				
	Adjusted HR	0.44	[0.27–0.71]	0.001
Healthcare costs				
	Adjusted mean difference (USD)	398.38	[-418.93–1,215.70]	0.339

CI: confidence interval; USD: United States dollars; HR: hazard ratios.

### Costs

The analysis of healthcare costs for the matched sample found a cost of USD 3,085.82 (95%CI 2,485.52–3,686.12) for the cohort of surgeries in hospitals with low surgical volume costs and USD 3,484.2 (95%CI 2,929.55–4,038.85) for the cohort in hospitals with a high surgical volume. However, these differences were not statistically significant (mean difference: 398.38; 95%CI −418.93 to 1,215.69; p=0.339) (Table 4).

## DISCUSSION

Based on administrative healthcare data, the present study identified the cohort of patients with gastric cancer who underwent gastrectomies in Bogota between 2014 and 2016, described the surgical volume of the hospitals where they underwent surgery, and determined how surgical volume was associated with 6-month survival and the healthcare costs paid by the Colombian health system. This study found that undergoing surgery in a hospital with a high surgical volume (26 or more gastrectomies per year) was associated with better 6-month survival than in a hospital with a low volume. No difference was found in the direct costs paid by the Colombian health system.

The findings of this investigation are consistent with several studies in the literature that report that surgical volume at hospitals where patients undergo surgery is associated with short-term mortality. In a systematic review by Mukai et al*.* of 23 studies with more than 1,000 patients each, the authors found that undergoing operations in hospitals with a high surgical volume was associated with lower postoperative mortality^18^. Of these studies, seven had more than 10,000 patients^6,11,13–15,23,25^. Additionally, Iwatsuki et al*.* evaluated 71,307 patients and found that undergoing operations in a hospital with 27 or more total gastrectomies per year resulted in lower 30-day postoperative mortality than a hospital with 11 or fewer cases per year (OR 0.53, 95%CI 0.43–0.63)^12^.

Most of the previously discussed studies used odds ratios as the effect estimator to compare the association of surgical volume with mortality. Only two studies used HR as the effect estimator, which was also used by our study. Anderson et al. reported an HR of 0.385 (p<0.001) when comparing hospitals with more than 30 surgeries per year with those with less than 10^2^. And Coupland et al*.* found an HR of 0.52 (p<0.0001) for hospitals with 80 or more surgeries per year compared to those with less than 20^8^.

With regard to the definition of high and low volume, the present study identified an inflection point for hospitals with 26 or more surgeries per year. This was determined by determining the relative risk of each possible cutoff point evaluated and identifying the lowest surgical volume value that was statistically <1. Then, the population was divided into two groups that were found to have significant differences in 30-day mortality according to a Poisson distribution. Different cutoff points have been used to define high and low surgical volume hospitals. Many studies use the observed distribution and some benchmarks, such as the 75th or 90th percentiles; others use prior definitions or administrative definitions^12,9,10^. The review by Mukai et al*.* presents a summary of the different cutoff points that have been used, which shows various values ranging from 6 or more to 80 or more^8,23^. However, most reports are between 13 and 30 surgeries per year.

In low-income countries, hospitals with higher surgical volume tend to have medical staff with more training or experience, and more advanced technology. While this could be related to higher patient care costs, the present study explored the relationship between hospital costs and surgical volume and did not find any statistically significant differences in the direct costs paid by the health system. Nevertheless, descriptively, the average cost was higher for patients who underwent surgery in hospitals with a high surgical volume. Murata *et* al. found that hospitals with a high surgical volume (more than 40 surgeries per year) were associated with shorter hospital stays and lower costs in Japan^19^.

The present study has several limitations. First, although the information in the study was taken from a highly standardized database with broad coverage, given that it is an administrative database, it does not contain relevant clinical information that could improve the estimates, for example, histologic type of cancer, anatomic location, extent of lymphatic dissection, and duration of surgery, among others. Second, it was not possible to study the number of surgeons or surgeon experience, which have been found to be associated with mortality. Third, no hospitals with annual surgical volumes between 20 and 25 surgeries were identified; therefore, these values could not be tested as cutoff points.

Nonetheless, the present study has several advantages. First, to our knowledge, this is the first study in a low- or middle-income country that presents information about hospitals with a high *versus* low volume of gastrectomies for cancer patients. This is very important for the development of policies for the country's health systems. Second, the administrative database that was used was highly standardized and came from the Colombian health system, which covers approximately 96% of the population of Bogota. Therefore, the estimate of the number of surgeries per hospital is very close to reality. While there could be some measurement problems in terms of patients who paid for gastrectomy, this is highly uncommon in Colombia, which has one of the lowest out-of-pocket expenses on the entire continent^17^. Third, the death certificates used to estimate the date of death contained high-quality data and had good coverage, and they enabled tracking patients regardless of the place of death (in another hospital or city other than Bogota). Fourth, the use of propensity scores to control for confounding and/or selection bias resulted in balance in the observable baseline characteristics, making the two groups comparable.

Finally, we consider this to be a pioneering study and one that is greatly important for analyzing the performance of health systems in low-income regions. The information available thus far has come from developed countries, and policies in low-income countries have been designed based on information from very different contexts. This study will provide local information for the design of policies in Colombia.

## CONCLUSION

This study demonstrates that in Bogota (Colombia), undergoing surgery in a hospital with a high surgical volume is associated with better 6-month survival and that there are no additional costs to the health system. Additionally, 26 or more gastrectomies per year were found to be a good cutoff point for defining a high surgical volume hospital.

## References

[1] Ajani JA, D'Amico TA, Bentrem DJ, Chao J, Cooke D, Corvera C (2022). Gastric Cancer, Version 2.2022, NCCN clinical practice guidelines in oncology. J Natl Compr Canc Netw.

[2] Anderson O, Ni Z, Møller H, Coupland VH, Davies EA, Allum WH (2011). Hospital volume and survival in oesophagectomy and gastrectomy for cancer. Eur J Cancer.

[3] Austin PC, Cafri G (2020). Variance estimation when using propensity-score matching with replacement with survival or time-to-event outcomes. Stat Med.

[4] Austin PC (2011). An Introduction to Propensity Score Methods for Reducing the Effects of Confounding in Observational Studies. Multivariate Behav Res.

[5] Austin PC (2009). Balance diagnostics for comparing the distribution of baseline covariates between treatment groups in propensity-score matched samples. Stat Med.

[6] Birkmeyer JD, Siewers AE, Finlayson EV, Stukel TA, Lucas FL, Batista I (2002). Hospital volume and surgical mortality in the United States. N Engl J Med.

[7] Castro JSL, Pelosof AG, Andrade-Cabral JGG, Seraphim AM, Taglieri E, Coimbra FJF (2022). Endoscopic characteristics of patients with complete pathological response after neoadjuvant chemotherapy for gastric and esophagogastric junction adenocarcinomas. Arq Bras Cir Dig.

[8] Coupland VH, Lagergren J, Lüchtenborg M, Jack RH, Allum W, Holmberg L (2013). Hospital volume, proportion resected and mortality from oesophageal and gastric cancer: a population-based study in England, 2004-2008. Gut.

[9] Dikken JL, Dassen AE, Lemmens VE, Putter H, Krijnen P, van der Geest L (2012). Effect of hospital volume on postoperative mortality and survival after oesophageal and gastric cancer surgery in the Netherlands between 1989 and 2009. Eur J Cancer.

[10] Dikken JL, van Sandick JW, Allum WH, Johansson J, Jensen LS, Putter H (2013). Differences in outcomes of oesophageal and gastric cancer surgery across Europe. Br J Surg.

[11] Ghaferi AA, Birkmeyer JD, Dimick JB (2011). Hospital volume and failure to rescue with high-risk surgery. Med Care.

[12] Iwatsuki M, Yamamoto H, Miyata H, Kakeji Y, Yoshida K, Konno H (2021). Association of surgeon and hospital volume with postoperative mortality after total gastrectomy for gastric cancer: data from 71,307 Japanese patients collected from a nationwide web-based data entry system. Gastric Cancer.

[13] Kuwabara K, Matsuda S, Fushimi K, Ishikawa KB, Horiguchi H, Fujimori K (2011). Quantitative assessment of the advantages of laparoscopic gastrectomy and the impact of volume-related hospital characteristics on resource use and outcomes of gastrectomy patients in Japan. Ann Surg.

[14] Learn PA, Bach PB (2010). A decade of mortality reductions in major oncologic surgery: the impact of centralization and quality improvement. Med Care.

[15] Lin HC, Xirasagar S, Lee HC, Chai CY (2006). Hospital volume and inpatient mortality after cancer-related gastrointestinal resections: the experience of an Asian country. Ann Surg Oncol.

[16] Lordick F, Carneiro F, Cascinu S, Fleitas T, Haustermans K, Piessen G (2022). Gastric cancer: ESMO Clinical Practice Guideline for diagnosis, treatment and follow-up. Ann Oncol.

[17] Mor N (2022). Lessons for developing countries from outlier country health systems. Front Public Health.

[18] Mukai Y, Kurokawa Y, Takiguchi S, Mori M, Doki Y (2017). Are treatment outcomes in gastric cancer associated with either hospital volume or surgeon volume?. Ann Gastroenterol Surg.

[19] Murata A, Muramatsu K, Ichimiya Y, Kubo T, Fujino Y, Matsuda S (2015). Influence of hospital volume on outcomes of laparoscopic gastrectomy for gastric cancer in patients with comorbidity in Japan. Asian J Surg.

[20] Nelen SD, Heuthorst L, Verhoeven RHA, Polat F, Kruyt PM, Reijnders K (2017). Impact of centralizing gastric cancer surgery on treatment, morbidity, and mortality. J Gastrointest Surg.

[21] Nobre KEL, Pereira MA, Ramos MFKP, Ribeiro U, Zilberstein B, Cecconello I (2021). Recurrence in PN0 gastric cancer: risk factors in the occident. Arq Bras Cir Dig.

[22] Richards N, Sorchik R, Brolan C (2018). CRVS development series: why the sustainable development goal agenda needs strong civil registration and vital statistics systems.

[23] Smith JK, McPhee JT, Hill JS, Whalen GF, Sullivan ME, Litwin DE (2007). National outcomes after gastric resection for neoplasm. Arch Surg.

[24] van Putten M, Nelen SD, Lemmens VEPP, Stoot JHMB, Hartgrink HH, Gisbertz SS (2018). Overall survival before and after centralization of gastric cancer surgery in the Netherlands. Br J Surg.

[25] Wainess RM, Dimick JB, Upchurch GR, Cowan JA, Mulholland MW (2003). Epidemiology of surgically treated gastric cancer in the United States, 1988-2000. J Gastrointest Surg.

